# Nrf2 is a potential prognostic marker and promotes proliferation and invasion in human hepatocellular carcinoma

**DOI:** 10.1186/s12885-015-1541-1

**Published:** 2015-07-21

**Authors:** Mingxin Zhang, Chao Zhang, Lingmin Zhang, Qi Yang, Suna Zhou, Qinsheng Wen, Jingjie Wang

**Affiliations:** 1 Department of Gastroenterology, Tangdu Hospital, Fourth Military Medical University, Xi’an, 710038 Shaanxi Province China; 2 Department of Anesthesiology, First Affiliated Hospital, Medical School, Xi’an Jiaotong University, Xi’an, 710061 Shaanxi Province China; 3 Department of Radiotherapy, Tangdu Hospital, Fourth Military Medical University, Xi’an, 710038 Shaanxi Province China

**Keywords:** Nuclear factor E2-related factor 2, Human hepatocellular carcinoma, Prognostic marker, Proliferation, Invasion

## Abstract

**Background:**

Nuclear factor E2-related factor 2 (Nrf2 or NFE2L2) is abundantly expressed in cancer cells and relates to proliferation, invasion, and chemoresistance. Our early observations also found that expression of Nrf2 was up-regulated in kinds of cancer including human hepatocellular carcinoma (HCC) cells. But there are limited reports about the expression, significance, function of Nrf2 in HCC.

**Methods:**

First, Nrf2 expression was analyzed in HCC cell lines and tumor samples. Then, the relationship of Nrf2 with clinicopathological factors and survival were analyzed. Further, the effect of Nrf2 on cell proliferation, apoptosis, and metastasis was examined *in vitro* by modulating expression of Nrf2 through specific shRNA or expression plasmid. Last, the potential mechanisms were also investigated.

**Results:**

Nrf2 was up-regulated in HCC, and expression of Nrf2 was correlated with tumor differentiation, metastasis, and tumor size. Univariate and multivariate analyses indicated that high Nrf2 expression might be a poor prognostic factor. Further studies demonstrated that inhibition of Nrf2 expression inhibited proliferation by inducing apoptosis and repressed invasion, and up-regulation of Nrf2 expression resulted in opposite phenotypes. Moreover, there are positive correlation between Nrf2 expression and that of Bcl-xL and MMP-9.

**Conclusion:**

Nrf2 is a potential prognostic marker and promotes proliferation and invasion in human hepatocellular carcinoma partly through regulating expression of Bcl-xL and MMP-9.

## Background

Hepatocellular carcinoma (HCC) is one of the most common malignancies worldwide, especially in Asia [1]. The mortality rate of HCC has been increasing in China since the 1990s, and HCC has become the second leading cause of cancer death [2]. Although there have been significant improvements in surgical techniques and diagnostic methods in recent years, long-term prognosis is still unsatisfactory largely due to the high recurrence and invasion rates even after resection (50 % to 70 % at 5 y) [3, 4]. Multiple risk factors have been associated with the initiation and development of HCC, including chronic infection of hepatitis viruses (B, C, or D), aflatoxin, alcohol abuse, hereditary metabolic liver diseases, and diabetes mellitus [5]. However, little is known regarding the molecular mechanisms underlying this aggressive behavior. Therefore, a reliable prognostic biomarker may help clinicians predict the characteristics of the malignancy and decrease the rate of unfavorable outcomes in a high-risk population.

Nuclear factor E2-related factor 2 (Nrf2 or NFE2L2) is a key transcription regulator for antioxidant and detoxification enzymes [6]. Nrf2 activation is observed in nonparenchymal cells including hepatic stellate cells and Kupffer cells as well as in parenchymal hepatocytes [7, 8]. Moreover, many kinds of Nrf2 target genes are also expressed in the liver. Nrf2 plays protective roles in hepatic inflammation, fibrosis, hepatocarcinogenesis, and regeneration via its target gene induction [9]. However, recent studies found that Nrf2 is abundantly expressed in cancer cells including HCC and relates to proliferation, invasion, and chemoresistance [10–12]. Our early observations also found that expression of Nrf2 was up-regulated in kinds of cancer including HCC [13–18]. But there are limited reports about the expression, significance, function of Nrf2 in HCC.

In this study, we investigated whether expression of Nrf2 level has prognostic significance in HCC. Immunohistochemical expression of Nrf2 was examined in a total of 65 HCC patients who underwent a surgical resection without any neoadjuvant or adjuvant chemotherapy. We also investigated whether the expression levels of Nrf2 correlate with malignant behaviors of HCC including proliferation, apoptosis, and invasion through modulation of Nrf2 expression by RNA interference and expression plasmid.

## Methods

### Patients

We chose80 patients received resection for HCC at Tangdu Hospital, Fourth Military Medical University and First Affiliated Hospital, Medical School, Xi’an JiaoTong University between January 2005 and December 2009. Of these, staging or clinicopathologic information was incomplete for 10 patients, and either specimen blocks or follow-up records were not available for 5 patients. As a result, 65 patients were retrospectively reviewed. None of these 65 patients received neoadjuvant or adjuvant chemotherapy before operation. Patients were followed closely until December 31, 2011 for more than 6 months, and the mean duration of follow-up was 16.6 months (±9.2 months). Tumor stage was defined according to tumor-node-metastasis (TNM) classification of the American Joint Committee on International Union against Cancer. Tumor differentiation was assessed according to Edmonson and Steiner grading system. The clinicopathological features of patients are shown in Table 1. Our study was approved by the ethics committee of the Fourth Military Medical University and written informed consents were obtained from all the patients.

**Table 1:** Clinicopathologic variables and the expression status of Nrf2

Variables	Total	Nrf2		*χ* ^2^	*P*
		High	Low		
Gender				0.828	0.381
Male	44	34	10		
Female	21	14	7		
Age				1.092	0.398
<60	30	24	6		
≥60	35	24	11		
Metastasis				15.023	<0.001
Negative	35	19	16		
Positive	30	29	1		
Differentiation				10.955	0.001
Well + Moderate	35	20	15		
Poor	30	28	2		
HBV infection				2.578	0.167
Negative	14	8	6		
Positive	51	40	11		
Liver cirrhosis				0.828	0.381
No	21	14	7		
Yes	44	34	10		
AFP				0.088	1.000
≤400 μg/L	21	16	5		
>400 μg/L	44	32	12		
Tumor size				5.388	0.026
<5 cm	34	21	13		
≥5 cm	31	27	4		
Tumor number				0.427	0.579
Single	35	27	8		
Multiple	30	21	9		

### Cell culture

HCC cell lines (Hep3B, Bel-7402, and HepG2) and normal liver cell line L02 were obtained from the Type Culture Collection Cell Bank, Chinese Academy of Science Committee (Shanghai, China). Cells were cultured in RPMI 1640 with 10 % of fetal bovine serum (FBS), 100 U/ml of penicillin, and 100 U/ml of streptomycin at 37 °C in a 5 % CO_2_ incubator.

### Immunohistochemical staining and analysis

Tissue specimens were fixed in neutral buffered formalin (10 % v/v formalin in water; pH 7.4) and embedded in paraffin wax. Serial sections of 4-μm thickness were cut and mounted on charged glass slides. Conditions for Nrf2 were optimized and evaluated by two independent pathologists. The rabbit polyclonal antibody against Nrf2 (Santa Cruz Biotechnology, Santa Cruz, CA) was used at dilutions of 1:500. The Streptavidin-Peroxidase technique (Golden Bridge International, Beijing, China) was used as described [13]. An irrelevant rabbit antiserum served as a negative control. Sections were counterstained with Mayer’s hematoxylin.

### Immunohistochemical analysis

Two observers who were blinded to clinical and follow-up data evaluated staining results independently and co-observed for a consensus when they were divergent. Each slide was evaluated using a semiquantitative scoring system for both the intensity of the stain and the percentage of positive malignant cells. Nrf2 immunoreactivity was predominant in the nucleus. The percentage scoring of immunoreactive tumor cells was as follows: 0 (0 %), 1 (1-10 %), 2 (11-50 %) and 3 (>50 %). The staining intensity was visually scored and stratified as follows: 0 (negative), 1 (weak), 2 (moderate) and 3 (strong). A final score was obtained for each case by multiplying the percentage and the intensity score. Therefore, tumors with a multiplied score exceeding 4 (median of total scores for Nrf2) were deemed to be high expressions of Nrf2; all other scores were considered to be low expressions of Nrf2 [13].

### Western blot analysis

Anti-Nrf2, anti-Bcl-xL, anti-MMP9, and anti-β-actin antibodies were obtained from Santa Cruz Biotech (Santa Cruz, CA, USA). For Western blot analyses, 20 μg of total protein were electrophoresed on a 10 % SDS-PAGE gel, transferred onto to PVDF membrane, blocked, and then incubated with primary antibody as indicated above. Corresponding horseradish peroxidase (HRP)-conjugated secondary antibody was then used on them at room temperature for 2 h. After chemiluminescence reaction with enhanced ECL detection reagents (Amersham, Little Chalfont, Buckinghamshire, England) according to the manufacturer’s instructions, the membranes were visualized by exposure to X-ray film in dark. Densitometric analysis was performed using Scion Image software (Scion Corporation, Frederick, MD).

### Quantitative real time polymerase chain reaction (qRT-PCR)

qRT-PCR assay was carried out by a BioRad iQ5 Real-Time PCR Detection System to analyze the mRNA levels of Nrf2. The reverse transcription reaction was carried out in a 20 μL volume with 1 μg total RNA. The reaction was incubated at 37 °C for 15 min, then 85 °C for 5 s; 1 μL of the RT product was used in each PCR. The PCR cycling began with template denaturation at 95 °C for 5 min, followed by 40 cycles of 95 °C for 10 s, 60 °C for 20 s, 72 °C for 20 s, and 78 °C for 20 s. Final PCR products were resolved in agarose gel electrophoresis and a single band of expected size indicated the specificity of the reaction. The PCR primer sets used for cDNA amplification were as follows: Nrf2 sense 5′-ACACGGTCCACAGCTCATC-3′, anti-sense 5′-TGCCTCCAAGTATGTCAATA -3′; and GAPDH sense 5′-ACCACAGTCCATGCCATCAC-3′, anti-sense 5′-TCCACCACC CTGTTGCTGTA-3′. Final PCR products were resolved in agarose gel electrophoresis and a single band of expected size indicated the specificity of the reaction. Relative quantification was performed using the 2^-ΔΔCT^, and data were normalized by using GAPDH as an internal standard. Each PCR amplification was performed in triplicate to verify the results.

### Immunofluorescence assay

Cells (5 × 10^4^ cells/mL) were grown on coverslips in 24-well plates and pretreated with different interventions. The cells were washed with cold PBS, fixed in 4 % paraformaldehyde, permeabilized with 0.3 % Triton X-100, blocked with 5 % bovine serum albumin (BSA), and incubated at 4 °C overnight with Nrf2 antibodies. After washing with PBS, cells were incubated at 37 °C for 1 h with FITC- conjugated secondary antibody, then stained with the fluorochrome dye DAPI (1 μg/ml, Roche) to visualize the cell nuclei, and observed under a fluorescence microscope (Olympus).

### shRNA design, plasmid construction and transfection

The pGP U6-shRNA plasmids were constructed by cloning the respective shRNAs into the pGPU6/GFP/Neo vector (GenePharma, Shanghai, China) and renamed as shRNA-Nrf2. shNC contained an unrelated shRNA sequence, with no homology to any human gene, and was used as a negative control. The sequence targeting Nrf2 were described before [16]. The primers for human Nrf2 cDNA were as follows: forward 5′-CCGCTCGAGATGATGGACTTGGAGCTGCC-3′, reverse 5′-GGGGTACCGTGTTTTTCTTAACATCTGGC-3′. Human Nrf2 cDNA was cloned into the cloning site of the vector pEGFP-N1 (GeneChem, Shanghai, China) using the standard recombinant DNA technique as described before [17]. The new plasmid was named as pEGFP-Nrf2. And a blank vector (pEGFP) was used as negative control. Cells were seeded in a 24-well plate at a concentration of 1 × 10^5^ cells per well. Lipofectamine 2000 (Invitrogen, Carlsbad, CA, USA) was used for transfection according to the manufacturer’s instructions. Fresh culture medium was changed 6 h after transfection, and the cells were harvested 48 h after transfection for analysis. The shNC was used as a negative control. For verification of knock-down or up-regulation of Nrf2 in the transient transfected cell line, qRT-PCR and western blot analysis were performed, with Nrf2 expression normalized to the control.

### Cell viability assays

Cell viability was determined using an MTT assay according to the manufacturer’s protocol. The absorbance of each well was measured using a multidetection microplate reader (BMG LABTECH, Durham, NC, USA) at a wavelength of 570 nm. All experiments were performed in quadruplicate.

### Cell apoptosis assays

Cells were washed with PBS and resuspended in 500 μL binding buffer containing 2.5 μL annexin V-phycoerythrin (PE) and 5 μL propidine iodide (PI) to determine the phosphatidylserine (PS) exposure on the outer plasma membrane. After incubation, the samples were analyzed using flow cytometry (FACSCalibur, BD Biosciences, San Jose, CA). The experiment was repeated three times.

### Cell invasion assay

Cell invasion was measured using transwell chambers (Millipore, Billerica, USA) coated with Matrigel. After transfection, the harvested cells were suspended in serum free RPMI 1640 and were added into the upper compartment of the chamber; conditioned RPMI 1640 medium with 20 % (v/v) FBS was used as a chemoattractant and placed in the bottom compartment of the chamber. After incubation, the cells were removed from the upper surface of the filter with a cotton swab. The invaded cells were then fixed and stained using 0.1 % crystal violet. The cells were quantified from five different fields under a light microscope. The experiment was repeated in triplicate.

### Statistical analysis

Statistical analysis was done using the SPSS software package (version 13.0, SPSS Institute). The association between staining index and other categorical factors potentially predictive of prognosis was analyzed using the Chi-square test and Fisher’s exact test. Overall survival (OS) was defined as the time from the date of surgery to the date of last follow-up or death from any case. Disease free survival (DFS) time was defined as the interval between the date of surgery and the date of recurrence. Survival curve and median survival were estimated by the Kaplan-Meier method. Their differences were verified by log-rank test. Multivariate analysis was done using the Cox proportional hazard regression analysis. Differences between groups were assessed using an unpaired, two-tailed Student’s *t* test; *P* < 0.05 was considered significant.

## Result

### Expression of Nrf2 in HCC tissues and its significance

**Fig. 1:**
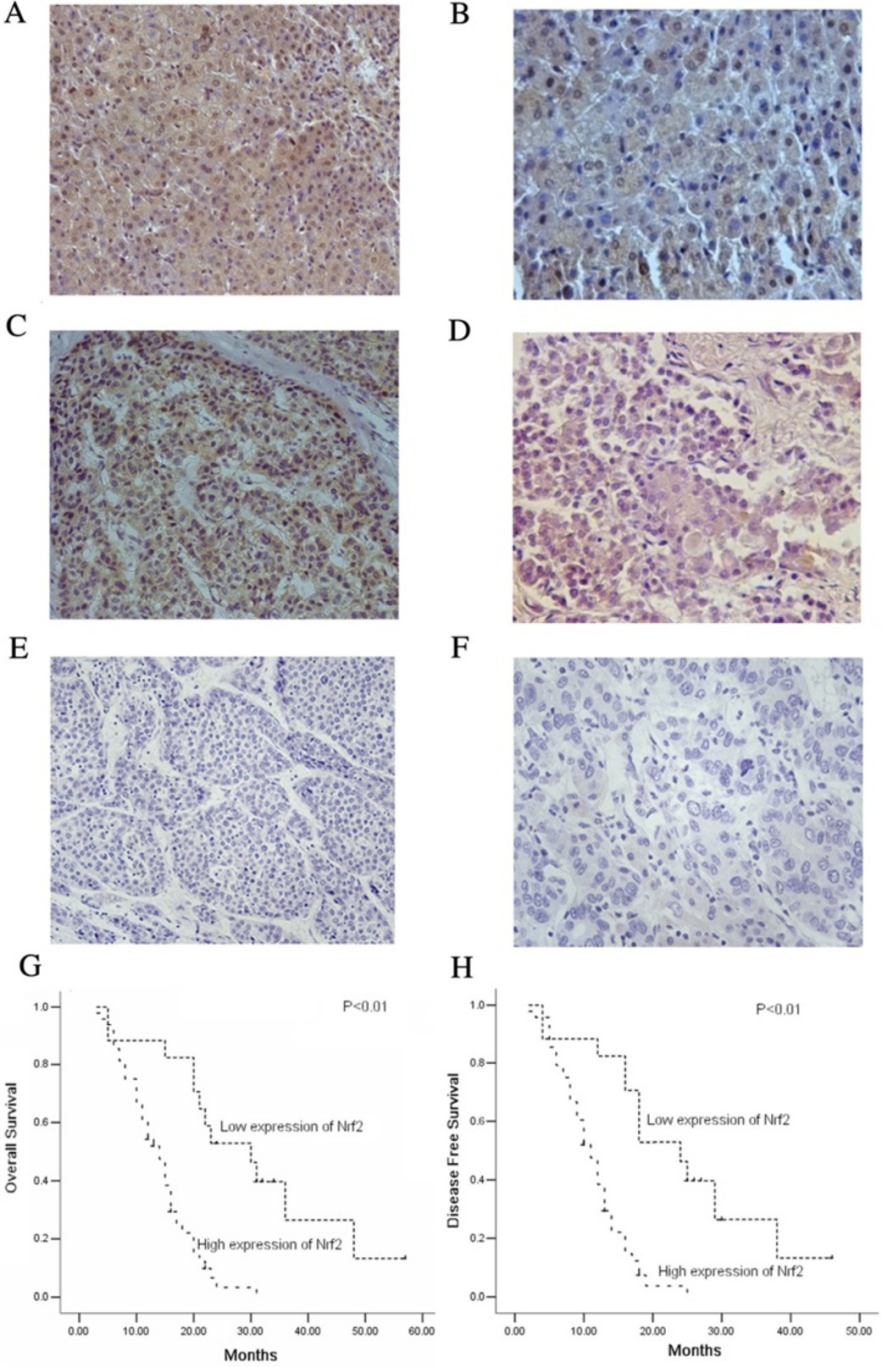
Expression and significance of Nrf2 in hepatocellular carcinoma. **a**-**d** Typical immunohistological features of Nrf2 expression in hepatocellular carcinoma (HCC). **a** Expression of Nrf2 in HCC with low differentiation. **b** Expression of Nrf2 in HCC with metastasis. **c** Expression of Nrf2 in HCC with well differentiation. **d** Expression of Nrf2 in HCC without metastasis. Magnifications: **a**, **c** × 200, **b**, **d** × 400; **e**-**f** Negative staining in hepatocellular carcinoma. Magnifications: **e**, × 200; **f**, × 400; **g**-**h** Kaplan-Meier survival analysis, P value was obtained using the log-rank test of the difference. **g** Overall survival (OS) differences between patients with high and low levels of Nrf2 protein expression; **h** Disease free survival (DFS) differences between patients with high and low levels of Nrf2 protein expression

Level of Nrf2 was evaluated by immunohistochemical analysis. Fig. 1a and d shows representative expression patterns of Nrf2 in HCC. Nrf2 was found nuclear and cytoplasmic localization, but primarily in the nucleus. And in HCC with poor differentiation or metastasis, Nrf2 showed more nuclear localization compared to that in HCC with well differentiation or no metastasis. There were significant correlations between the high level of Nrf2 expression and the tumor differentiation, metastasis, and tumor size,. However, the high level rates were not significantly correlated with gender, age, HBV infection, liver cirrhosis. alpha-fetal protein (AFP) levels, and tumor number (Table 1). Then, Kaplan-Meier analysis was used to calculate the impact of classic clinicopathologic features and protein expression on survival (Table 2, Fig. 1g and h). High expression of Nrf2, tumor differentiation, and metastasis were associated with decreased survival (*P* < 0.05), whereas other clinicopathological variables were not significant. Cox regression analysis revealed a statistically significant correlation with Nrf2 expression (*P* < 0.05, Table 3).

**Table 2:** Univariate analysis for overall survival and disease free survival

Variables	Overall survival		*P*	Disease free survival		*P*
	Median ± SE	95 % CI		Median ± SE	95 % CI	
Nrf2			<0.01			<0.01
Low	30.40 ± 4.16	22.25-38.56		24.43 ± 3.33	17.90-30.96	
High	13.87 ± 0.95	12.02-15.73		11.24 ± 0.76	9.75-12.73	
Gender			0.93			0.91
Male	17.56 ± 1.32	14.97-20.15		15.43 ± 2.91	9.72-21.13	
Female	19.07 ± 3.64	11.94-26.20		14.15 ± 1.05	12.08-16.22	
Age			0.94			0.91
<60	18.98 ± 2.50	14.08-23.88		15.33 ± 1.99	11.43-19.23	
≥60	18.43 ± 2.31	13.90-22.97		14.93 ± 1.86	11.29-18.56	
Metastasis			<0.01			<0.01
Negative	22.56 ± 2.48	17.69-27.42		18.18 ± 1.99	14.29-22.07	
Positive	12.97 ± 1.18	10.66-15.29		10.47 ± 0.93	8.64-12.30	
Differentiation			0.01			0.02
Well + Moderate	22.13 ± 2.52	17.20-27.07		17.81 ± 2.01	13.87-21.75	
Poor	13.51 ± 1.22	11.12-15.89		10.94 ± 0.92	9.02-12.87	
HBV infection			0.69			0.70
Negative	19.64 ± 3.90	12.00-27.29		15.89 ± 3.07	9.87-21.92	
Positive	18.80 ± 2.02	14.83-22.77		15.24 ± 1.65	12.01-18.47	
Liver cirrhosis			0.09			0.10
No	21.09 ± 2.22	16.73-25.44		17.09 ± 1.77	13.62-20.56	
Yes	17.24 ± 2.13	13.07-21.40		13.90 ± 1.70	10.56-17.23	
AFP			0.20			0.19
≤400 μg/L	20.84 ± 2.02	16.88-24.79		16.82 ± 1.62	13.65-19.99	
>400 μg/L	17.78 ± 2.27	13.35-22.22		14.36 ± 1.81	10.80-17.91	
Tumor size			0.09			0.10
<5 cm	21.98 ± 2.77	16.55-27.41		17.72 ± 2.22	13.38-22.06	
≥5 cm	15.25 ± 1.56	12.18-18.31		12.35 ± 1.27	9.85-14.85	
Tumor number			0.60			0.57
Single	20.21 ± 2.71	14.89-25.52		16.35 ± 2.19	12.06-20.63	
multiple	16.83 ± 1.79	13.32-20.35		13.63 ± 1.43	10.84-16.43

**Table 3:** Multivariate Cox proportional hazards analysis for overall survival and disease free survival

Variables	Overall survival		*P*	Disease free survival		*P*
	RR	95 % CI		RR	95 % CI	
Nrf2	5.96	2.46-14.69	<0.01	5.84	2.37-14.39	<0.01
Gender	0.62	0.30-1.27	0.20	0.63	0.31-1.29	0.20
Age	0.85	0.23-3.14	0.81	0.86	0.23-3.17	0.82
Metastasis	0.96	0.23-4.07	0.96	1.08	0.27-4.32	0.92
Differentiation	0.76	0.16-3.76	0.74	0.67	0.14-3.16	0.62
HBV infection	0.64	0.29-1.40	0.26	0.64	0.28-1.41	0.26
Liver cirrhosis	1.78	0.90-3.51	0.10	1.80	0.92-3.55	0.09
AFP	1.93	0.92-4.06	0.08	1.91	0.91-4.01	0.09
Tumor size	1.56	0.57-4.24	0.39	1.59	0.58-4.31	0.39
Tumor number	1.73	0.45-6.66	0.43	1.72	0.45-6.60	0.43

### Expression and subcellular location of Nrf2 in HCC cell lines

**Fig. 2:**
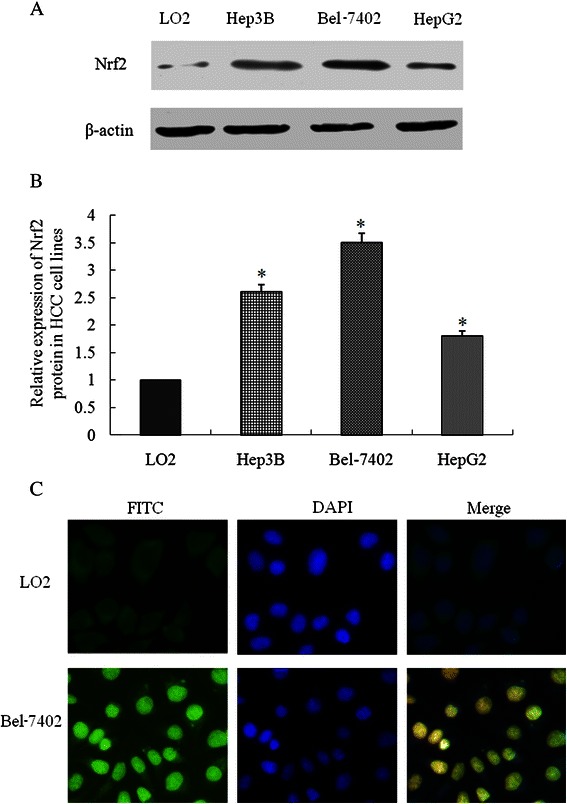
Expression and Subcellular location of Nrf2 in hepatocellular carcinoma cell lines. **a**-**b** Expression of Nrf2 in different human hepatocellular carcinoma cell lines (Hep3B, Bel-7402, and HepG2), with normal human liver cell line LO2 as control; **c** Subcellular location of Nrf2 was detected by immunofluorescence assay. In LO2 cells, Nrf2 expression was present in the cytoplasm, while in Bel-7402 cells, Nrf2 localization was found both in nucleus and cytoplasm, but mainly in nucleus. Magnifications: ×400. *P <0.05 compared with LO2

Since high level of Nrf2 expression correlated with the tumor differentiation, metastasis, and tumor size and served as independent prognostic factor, we then investigate the expression of Nrf2 in HCC cell lines. After detection of expression of Nrf2 by western blot, all HCC cell lines (Hep3B, Bel-7402, and HepG2) had an over-expression of Nrf2 compared to normal liver cell line L02 (Fig. 2a). Bel-7402 and HepG2, with highest or lowest expression levels of Nrf2, were chose for further experiments. Then, subcellular location of Nrf2 was evaluated by immunofluorescence assay. In LO2 cells, Nrf2 expression was present in the cytoplasm, while in Bel-7402 cells, Nrf2 localization was found both in nucleus and cytoplasm, but mainly in nucleus (Fig. 2b). The subcellular location of Nrf2 in Bel-7402 was consistent with that of immunohistochemical results.

### Transient transfection effect on Nrf2 mRNA and protein level

**Fig. 3:**
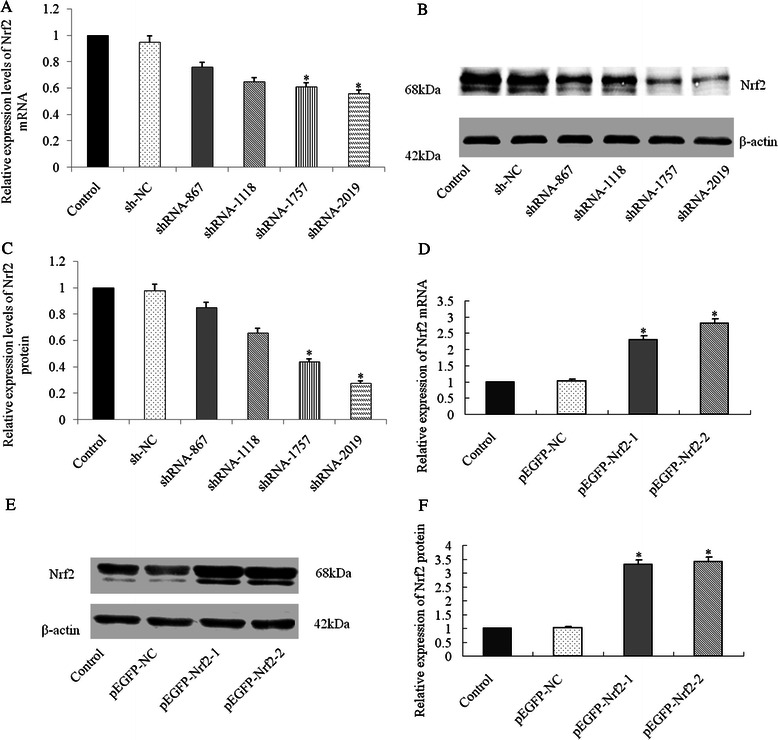
Modulation of endogenous Nrf2 expression. **a** After transfected with Nrf2-shRNA (shRNA-867, shRNA-1118, shRNA-1757, or shRNA-2019) or control shRNA (shNC), expression levels of Nrf2 mRNA in Bel-7402 cells were detected by qRT-PCR; **b**-**c** After transfected with Nrf2-shRNA (shRNA-867, shRNA-1118, shRNA-1757, or shRNA-2019) or control shRNA (shNC), expression levels of Nrf2 protein in Bel-7402 cells were detected by western blot; **d** After transfected with Nrf2 expression plasmid (pEFGP-Nrf2-1 or pEFGP-Nrf2-2) or mock pEGFP plasmid (pEGFP-NC), expression levels of Nrf2 mRNA in HepG2 cells were detected by qRT-PCR; **e**-**f** After transfected with Nrf2 expression plasmid (pEFGP-Nrf2-1 or pEFGP-Nrf2-2) or mock pEGFP plasmid (pEGFP-NC), expression levels of Nrf2 protein in HepG2 cells were detected by western blot. *P <0.05 compared with control (Bel-7402 cells or HepG2 cells respectively) or shNC and pEGFP-NC

To knock down the endogenous expression of Nrf2 in Bel-7402 cells, we applied a plasmid vector expressing specific shRNA sequence targeting Nrf2 (shRNA-Nrf2). As a control, we stably transfected the Bel-7402 cells with the same plasmid vector expressing a control shRNA sequence (shNC) that did not target any known human gene. Through mRNA and protein expression analysis, we found that the shNC cells have a similar Nrf2 level as the parental Bel-7402 cells, which were significantly higher than the level in the shNrf2 cells (Fig. 3a, b and c). We then applied a expression plasmid named pEGFP-Nrf2 to up-regulate expression of Nrf2 in HepG2. The mRNA and protein expression analysis confirmed that pEGFP-Nrf2 significantly increased expression of Nrf2 in transfected HepG2 cells (Fig. 3d, e and f).

### Nrf2 promotes cell proliferation by inhibiting apoptosis

**Fig. 4:**
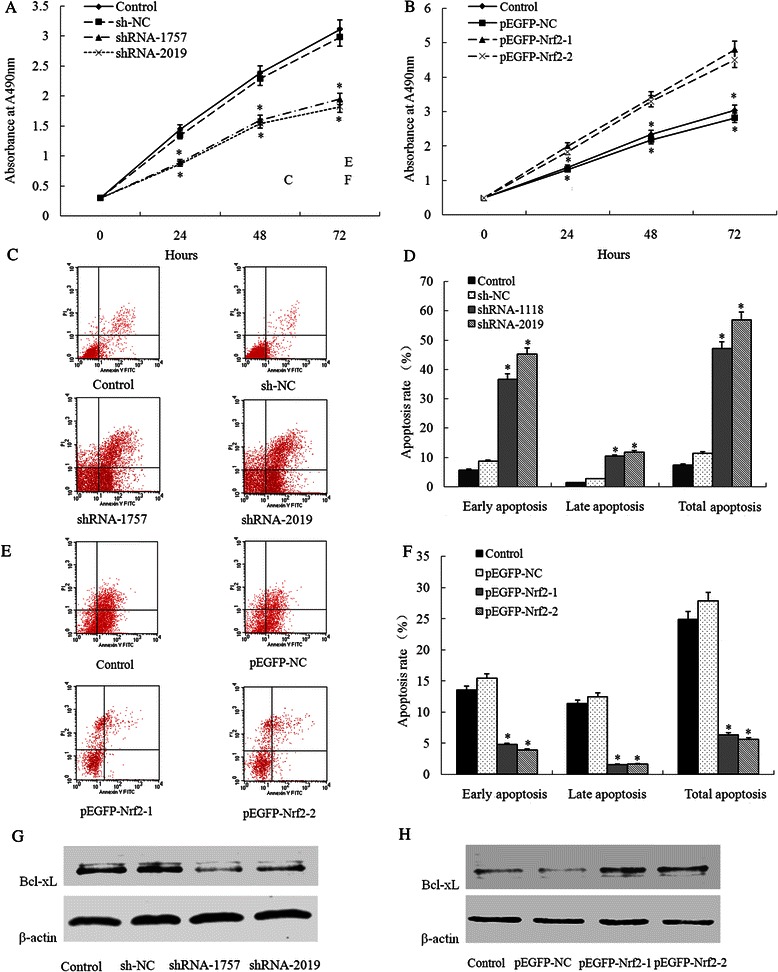
Effect of Nrf2 on cell proliferation and apoptosis. **a** After shRNA-Nrf2 (shRNA-1757 or shRNA-2019) or control shRNA (shNC) transduction, the growth of Bel-7402 cells was analyzed at different time points using the MTT assay; **b** After Nrf2 expression plasmid (pEFGP-Nrf2-1 or pEFGP-Nrf2-2) or mock pEGFP plasmid (pEGFP-NC) transduction, the growth of HepG2 cells was analyzed at different time points using the MTT assay; **c**-**d** Flow cytometric analysis of the effect of Nrf2 on the apoptosis of Bel-7402 cells by down-regulation of expression of Nrf2; **e**-**f** Flow cytometric analysis of the effect of Nrf2 on the apoptosis of HepG2 cells by up-regulation of expression of Nrf2. **g** After shRNA-Nrf2 (shRNA-1757 or shRNA-2019) or control shRNA (shNC) transduction, expression of Bcl-xL were detected by western blot in Bel-7402 cells; **h** After Nrf2 expression plasmid (pEFGP-Nrf2-1 or pEFGP-Nrf2-2) or mock pEGFP plasmid (pEGFP-NC) transduction, expression of Bcl-xL were detected by western blot in HepG2 cells. *P < 0.05 compared with control (Bel-7402 cells or HepG2 cells respectively) or shNC and pEGFP-NC

To investigate whether Nrf2 modulates cell proliferation in HCC cells, we assayed its effect on cell proliferation activity. The proliferation activities of Bel-7402 cells transfected with shRNA-Nrf2 and HepG2 cells transected with pEGFP-Nrf2 were determined using an MTT assay. As shown in Fig. 4a and b, inhibition of Nrf2 expression had a significant decrease in cell viability while increasing Nrf2 expression got the opposite results (*P* < 0.05). Following experiments demonstrated that shRNA-Nrf2 transfection induced apoptosis and pEGFP-Nrf2 transfection inhibited apoptosis, showing that the cell proliferation inhibition effect was partly due to the inhibition of apoptosis (Fig. 4c to f). We therefore assessed the expression of Bcl-xL, an apoptosis related protein regulating death and survival, in Bel-7402 cells transfected with shRNA-Nrf2 and HepG2 cells transected with pEGFP-Nrf2. Expression of Bcl-xL was positively correlated with the expression of Nrf2: inhibition of Nrf2 decreased the Bcl-xL expression while up-regulation of Nrf2 increased the Bcl-xL expression (Fig. 4g to h).

### Nrf2 regulates cell invasion *in vitro*

**Fig. 5:**
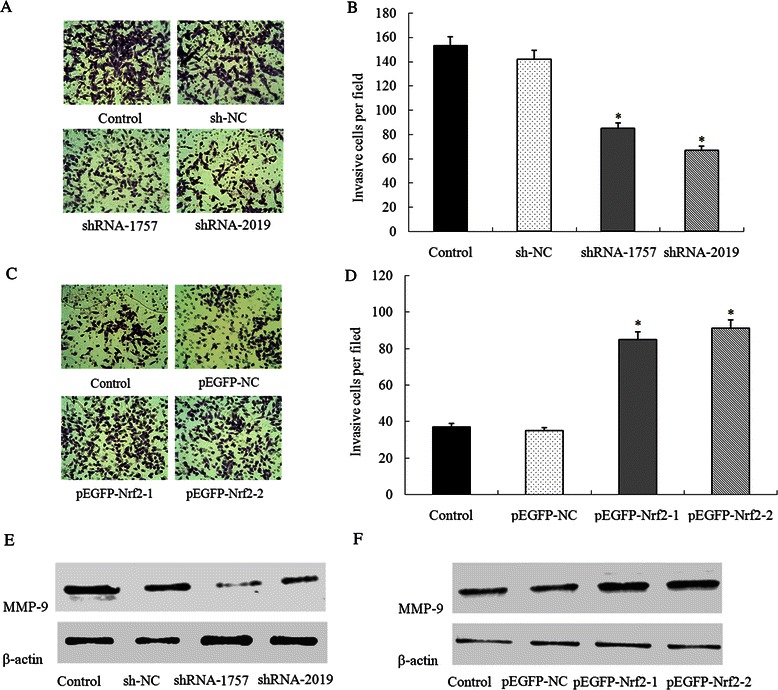
Effect of Nrf2 on cell invasion *in vitro.* **a** Bel-7402 cells transfected with shRNA-Nrf2 (shRNA-1757 or shRNA-2019) or control shRNA (shNC) were subjected to transwell invasion assays; **b** The invasive cell numbers are the average count of five random microscopic fields detected using the transwell invasion assay; **c** HepG2 cells transfected with Nrf2 expression plasmid (pEFGP-Nrf2-1 or pEFGP-Nrf2-2) or mock pEGFP plasmid (pEGFP-NC) were subjected to transwell invasion assays; **d** The invasive cell numbers are the average count of five random microscopic fields detected using the transwell invasion assay. Each bar represents the mean ± SD of the counts. **e** After shRNA-Nrf2 (shRNA-1757 or shRNA-2019)) or control shRNA (shNC) transduction, expression of MMP-9 were detected by western blot in Bel-7402 cells; **f** After Nrf2 expression plasmid (pEFGP-Nrf2-1 or pEFGP-Nrf2-2) or mock pEGFP plasmid (pEGFP-NC) transduction, expression of MMP-9 were detected by western blot in HepG2 cells. *P < 0.05 compared with control (Bel-7402 cells or HepG2 cells respectively) or shNC and pEGFP-NC

Because there was a correlation between Nrf2 and metastasis, a transwell assay was performed to investigate the role of Nrf2 on the invasion of HCC cells. Down-regulation of Nrf2 expression repressed the cell invasion ability of Bel-7402 cells, and up-regulation of Nrf2 expression promoted the cell invasion ability of HepG2 cells (*P* < 0.05, Fig. 5a to d). These findings suggest that Nrf2 regulates cell invasion of the HCC cell lines *in vitro*. We therefore assessed the expression of matrix metalloproteinases-9 (MMP-9), a protein regulating cell migration and invasion, in Bel-7402 cells transfected with shRNA-Nrf2 and HepG2 cells transected with pEGFP-Nrf2. Expression of MMP-9 was positively correlated with the expression of Nrf2: inhibition of Nrf2 decreased the MMP-9 expression while up-regulation of Nrf2 increased the MMP-9 expression (Fig. 5e to f).

## Discussion

Nrf2, a key transcription factor, plays a pivotal role in endogenous protection against oxidative stress. Upon exposure of cells to oxidative stress or chemopreventive compounds, Nrf2 translocates to the nucleus, forms a heterodimer with its obligatory partner Maf, and binds to the antioxidant response element (ARE) sequence to activate those encoding endogenous antioxidants, phase II detoxifying enzymes, and transporters [19]. As a result, activation of the Nrf2 pathway confers protection against subsequent toxic/carcinogenic exposure. Therefore, Nrf2 has been viewed as a “good” protein that protects humans from genotoxic damage caused by carcinogens. Several *in vivo* studies using Nrf2-null mice further verified the pivotal role of Nrf2 in cancer protection [20–22].

Interestingly, recent emerging data has revealed the “dark” side of Nrf2. Nrf2 and its downstream genes are over-expressed in many cancer cell lines and human cancer tissues, giving cancer cells an advantage for survival and growth [23]. In cancer tissues and cells, loss of Keap1, an Nrf2 negatively regulator, leads to nuclear localization and constitutive activation of Nrf2 [24–27]. Furthermore, Nrf2 is up-regulated in resistant cancer cells and is thought to be responsible for acquired chemoresistance. Observations including ours found that mutation or over-expression of Nrf2 in kinds of cancer [13–18]. Our previous studies suggest that Nrf2 confers chemoresistance of HCC and inhibition of Nrf2 by sorafenib could sensitize Bel-7402/5-FU cells to 5-FU [17]. But there are limited reports about the expression, significance, function of Nrf2 in HCC. Then, we will focus our attention on the oncogenic functions of Nrf2 in HCC in the certain research.

Our results showed that there were significant correlations between the expression of Nrf2 and metastasis, differentiation, and tumor size. Then, high expression level of Nrf2 was an independent factor that indicated poor prognosis in HCC patients. Furthermore, we detected the expression and subcellular localization of Nrf2 in HCC cell lines. Consistent with the immunohistochemical results and other reports in lung cancer and cervical cancer, Nrf2 were over-expression and nuclear localization in HCC, indicating Nrf2 was constitutive activated [26, 27]. This evidence suggests that over-expression of Nrf2 in tumor cells may play roles in the development of HCC and may have prognostic value.

Furthermore, to reveal the exact role of Nrf2 in HCC, we tested the effect of Nrf2 on proliferation, apoptosis, and invasion by modulating the expression level of Nrf2 using Nrf2-shRNA and pEGFP-Nrf2. The results suggested that Nrf2 acted as an oncogene in HCC. First, Nrf2 could induce proliferation due to the regulation of apoptosis and promote invasion in HCC cells. In our opinion, this invasion related ability could reveal the correlation between Nrf2 and metastasis: over-expression of Nrf2 promoted the metastasis of HCC cells. Then, we investigated the potential mechanisms of Nrf2 in regulating proliferation, apoptosis, and invasion. Bcl-2 family proteins are the prototypical antiapoptotic proteins, and Bcl-xL was the first protein discovered with a similar function [28]. MMP9, which belongs to the ECM-degrading enzyme family, is involved in migration and invasion of tumor cells [29]. Considering the role of Bcl-xL and MMP-9 in cell survival and invasion respectively, we investigated their relationship with expression of Nrf2. There are positive correlation between Nrf2 expression and that of Bcl-xL and MMP-9. Consistent with previous studies, Nrf2 up-regulated expression of Bcl-xL and MMP-9 in HCC cells resulted in cell proliferation, apoptosis inhibitation, and invasion [17, 30]. We will carry out *in vivo* experiments further to confirm the role of Nrf2 and its target genes in HCC.

## Conclusions

In conclusion, this was the first study to systemically evaluate the oncogenic functions of the Nrf2 in HCC. Our findings demonstrated that Nrf2 was up-regulated in HCC, and expression of Nrf2 was correlated with tumor differentiation metastasis, and tumor size. We found that Nrf2 was an independent prognostic factor in HCC patients. We also concluded that Nrf2 promoted proliferation by inhibiting apoptosis and enhanced the invasive ability of HCC cells partly through regulating expression of Bcl-xL and MMP-9.

## Abbreviations

Nrf2: Nuclear factor E2-related factor 2
HCC: Human hepatocellular carcinoma
TNM: Tumor-nodemetastasis
HRP: Horseradish peroxidase
qRT-PCR: Quantitative real time polymerase chain reaction
PI: Propidine iodide
PS: Phosphatidylserine
AFP: Alpha-fetal protein

## References

[1] Schütte K, Bornschein J, Malfertheiner P (2009). Hepatocellular carcinoma--epidemiological trends and risk factors. Dig Dis.

[2] He J, Gu D, Wu X, Reynolds K, Duan X, Yao C (2005). Major causes of death among men and women in China. N Engl J Med.

[3] Cabrera R, Nelson DR (2010). Review article: the management of hepatocellular carcinoma. Aliment Pharmacol Ther.

[4] Cormier JN, Thomas KT, Chari RS, Pinson CW (2006). Management of hepatocellular carcinoma. J Gastrointest Surg.

[5] El-Serag HB (2011). Hepatocellular carcinoma. N Engl J Med.

[6] Surh YJ, Kundu JK, Li MH, Na HK, Cha YN (2009). Role of Nrf2-mediated heme oxygenase-1 upregulation in adaptive survival response to nitrosative stress. Arch Pharm Res.

[7] Vasiliou V, Qamar L, Pappa A, Sophos NA, Petersen DR (2003). Involvement of the electrophile responsive element and p53 in the activation of hepatic stellate cells as a response to electrophile menadione. Arch Biochem Biophys.

[8] Yeligar SM, Machida K, Kalra VK (2010). Ethanol-induced HO-1 and NQO1 are differentially regulated by HIF-1alpha and Nrf2 to attenuate inflammatory cytokine expression. J Biol Chem.

[9] Shin SM, Yang JH, Ki SH (2013). Role of the Nrf2-ARE pathway in liver diseases. Oxid Med Cell Longev.

[10] Gao AM, Ke ZP, Shi F, Sun GC, Chen H (2013). Chrysin enhances sensitivity of BEL-7402/ADM cells to doxorubicin by suppressing PI3K/Akt/Nrf2 and ERK/Nrf2 pathway. Chem Biol Interact.

[11] Gao AM, Ke ZP, Wang JN, Yang JY, Chen SY, Chen H (2013). Apigenin sensitizes doxorubicin-resistant hepatocellular carcinoma BEL-7402/ADM cells to doxorubicin via inhibiting PI3K/Akt/Nrf2 pathway. Carcinogenesis.

[12] Lee SE, Yang H, Jeong SI, Jin YH, Park CS, Park YS (2012). Induction of heme oxygenase-1 inhibits cell death in crotonaldehyde-stimulated HepG2 cells via the PKC-δ-p38-Nrf2 pathway. PLoS One.

[13] Wang J, Zhang M, Zhang L, Cai H, Zhou S, Zhang J (2010). Correlation of Nrf2, HO-1, and MRP3 in gallbladder cancer and their relationships to clinicopathological features and survival. J Surg Res.

[14] Ma R, Zhang M, Wang J, Cai H, Yeer M, Duan X (2011). Expression and distribution of Nrf2 in several hepatocellular carcinoma cell lines. Xi Bao Yu Fen Zi Mian Yi Xue Za Zhi.

[15] Mao J, Tangsakar E, Shen H, Wang Z, Zhang M, Chen J (2011). Expression and clinical significance of Nrf2 in esophageal squamous cell carcinoma. Xi Bao Yu Fen Zi Mian Yi Xue Za Zhi.

[16] Zhang L, Wang N, Zhou S, Ye W, Jing G, Zhang M (2012). Propofol induces proliferation and invasion of gallbladder cancer cells through activation of Nrf2. J Exp Clin Cancer Res.

[17] Pan H, Wang H, Zhu L, Mao L, Qiao L, Su X (2013). The role of Nrf2 in migration and invasion of human glioma cell U251. World Neurosurg.

[18] Zhou S, Ye W, Shao Q, Zhang M, Liang J (2013). Nrf2 is a potential therapeutic target in radioresistance in human cancer. Crit Rev Oncol Hematol.

[19] Ma Q (2013). Role of nrf2 in oxidative stress and toxicity. Annu Rev Pharmacol Toxicol.

[20] Kwak MK, Itoh K, Yamamoto M, Sutter TR, Kensler TW (2001). Role of transcription factor Nrf2 in the induction of hepatic phase 2 and antioxidative enzymes *in vivo* by the cancer chemoprotective agent, 3H-1, 2-dimethiole-3-thione. Mol Med.

[21] Chan JY, Kwong M (2000). Impaired expression of glutathione synthetic enzyme genes in mice with targeted deletion of the Nrf2 basic-leucine zipper protein. Biochim Biophys Acta.

[22] Chanas SA, Jiang Q, McMahon M, McWalter GK, McLellan LI, Elcombe CR (2002). Loss of the Nrf2 transcription factor causes a marked reduction in constitutive and inducible expression of the glutathione S-transferase Gsta1, Gsta2, Gstm1, Gstm2, Gstm3 and Gstm4 genes in the livers of male and female mice. Biochem J.

[23] Zhou S, Ye W, Zhang M, Liang J (2012). The effects of nrf2 on tumor angiogenesis: a review of the possible mechanisms of action. Crit Rev Eukaryot Gene Expr.

[24] Kim WD, Kim YW, Cho IJ, Lee CH, Kim SG (2012). E-cadherin inhibits nuclear accumulation of Nrf2: implications for chemoresistance of cancer cells. J Cell Sci.

[25] Kawai Y, Garduño L, Theodore M, Yang J, Arinze IJ (2011). Acetylation-deacetylation of the transcription factor Nrf2 (nuclear factor erythroid 2-related factor 2) regulates its transcriptional activity and nucleocytoplasmic localization. J Biol Chem.

[26] Ohta T, Iijima K, Miyamoto M, Nakahara I, Tanaka H, Ohtsuji M (2008). Loss of Keap1 function activates Nrf2 and provides advantages for lung cancer cell growth. Cancer Res.

[27] Konstantinopoulos PA, Spentzos D, Fountzilas E, Francoeur N, Sanisetty S, Grammatikos AP (2011). Keap1 mutations and Nrf2 pathway activation in epithelial ovarian cancer. Cancer Res.

[28] Boise LH, González-García M, Postema CE, Ding L, Lindsten T, Turka LA (1993). bcl-x, a bcl-2-related gene that functions as a dominant regulator of apoptotic cell death. Cell.

[29] Bauvois B (2012). New facets of matrix metalloproteinases MMP-2 and MMP-9 as cell surface transducers: outside-in signaling and relationship to tumor progression. Biochim Biophys Acta.

[30] Niture SK, Jaiswal AK (2013). Nrf2-induced antiapoptotic Bcl-xL protein enhances cell survival and drug resistance. Free Radic Biol Med.

